# The Evaluation of a Modified Decision Aid for Older Women With Low Health Literacy

**DOI:** 10.1093/geroni/igaa057.1397

**Published:** 2020-12-16

**Authors:** Tamara Cadet, Mara Schonberg

**Affiliations:** 1 Simmons University, Boston, Massachusetts, United States; 2 Beth Israel Deaconess Medical Center, Boston, Massachusetts, United States

## Abstract

Given the lack of evidence recommending mammography for women >75 years, guidelines recommend that older women be informed of the uncertainty of benefits and of potential harms. The objective for this study was to evaluate the effect of a mammography decision aid (DA) designed for older women with low health literacy (LHL) on their decisional conflict and knowledge of mammography’s benefits and harms in a pretest-posttest trial. Women, 75-89 years were eligible for this study if they had not had a screening mammogram in six months, a history of breast cancer/dementia and had LHL (defined as reporting difficulty completing medical forms on one’s own or obtaining < college education). Forty-four women participated. their mean age was 78, 74% had a high school degree or less, and 53% were non-Hispanic White. Overall, women reported that the DA helped them prepare to talk with their clinician quite a bit (Mean =3.6/5.0 on preparation for decision making scale) and 97% found the DA helpful. Using McNemar’s test, decisional conflict did not change and knowledge on a 10 item true/false test on mammography screening did not change; however, after receiving the DA women were correctly less likely to think that having a mammogram would prevent cancer. With the shift toward shared decision-making for women > 75 years, there is a need to engage women of all literacy levels to make these decisions and we have developed a DA on mammography screening for older women with LHL that these women perceive to be helpful.

